# Luteolin-7-O-glucoside from *Elsholtzia ciliata* extract inhibits the replication of coronavirus

**DOI:** 10.1371/journal.pone.0325371

**Published:** 2025-06-03

**Authors:** Siyun Lee, Jang Hoon Kim, Chansoo Kim, Woochul Jung, Jayhyun Park, Junsoo Park

**Affiliations:** 1 Division of Biological Science and Technology, Yonsei University, Wonju, Republic of Korea,; 2 Department of Herbal Crop Research, National Institute of Horticultural & Herbal Science, RDA, Eumsung, Republic of Korea; 3 R&D team, Korea Mine Rehabilitation and Mineral Resources Corporation, Wonju, Korea; Nazarbayev University School of Medicine, PAKISTAN

## Abstract

Coronaviruses are RNA viruses with a high mutation rate that cause many human diseases, from severe COVID-19 to mild common cold. Therefore, discovering various medicines is required to reduce the symptoms of coronavirus infection. This report showed that the ethanol extract of the aerial parts of *Elsholtzia ciliata* (ECE) and its constituents can inhibit HCoV-OC43 (Human Coronavirus OC43) replication. HCoV-OC43, a human betacoronavirus genetically related to SARS-CoV-2, was used as a surrogate virus under BSL-2 conditions. To evaluate the antiviral properties of ECE, viral RNA levels were quantified using qRT-PCR, and viral protein expression was analyzed through Western blotting. ECE reduced coronavirus-induced plaque formation and viral RNA and protein expression in coronavirus-infected cells and conditioned media. Additionally, this was confirmed to have an inhibitory activity on virus production and improved cytopathic effects. ECE showed no cytotoxicity up to 40 µg/mL in vitro. As *Elsholtzia ciliata* has traditionally been consumed as a tea, oral administration could be a suitable route for further in vivo investigation. The main components of ECE were revealed by HPLC analysis and were isolated into four single compounds **1**–**4**. Among them, luteolin-7-*O*-glucoside was effective in inhibiting the replication of coronavirus. Luteolin-7-O-glucoside demonstrated greater antiviral activity than ECE, with an estimated IC_50_ of 2 µM (around 1 µg/mL) compared to 5 µg/mL for ECE. This finding suggests that luteolin-7-O-glucoside could be a key contributor to the antiviral activity of the ECE. Finally, these results collectively suggest that *Elsholtzia ciliata* can be used as a potential antiviral treatment.

## Introduction

Coronavirus contributes to various diseases, from mild common cold to emergent COVID-19, and approximately 15−30% of common cold cases are associated with human coronavirus infection [1,2]. Four human coronaviruses (HCoV-229E, HCoV-NL63, HCoV-OC43, and HCoV-HKU1) are global endemic and contribute to the common cold (upper respiratory tract infection) in adults [3,4]. Among them, HCoV-OC43 and SARS-CoV-2 belong to beta coronavirus and HCoV-OC43 is commonly used to study SARS-CoV-2 [5–7]. Because coronavirus is an RNA virus, the coronavirus genome is unstable, unlike DNA viruses, and tends to evolve quickly due to its high mutation rate [8,9]. Although the vaccines against SARS-CoV-2 are developed, the novel variants can be produced via an immune evasion mechanism, and they are expected to infect humans repeatedly, like seasonal common cold [10]. For this reason, safe and effective medicines against coronavirus should be developed to control coronavirus-related diseases.

*Elsholtzia ciliata* (Thunb.) Hyl. belongs to the genus *Elsholtzia* and the family Lamiaceae [11]. Plants of the *Elsholtzia* genus have been traditionally used to treat colds, fever, and pneumonia [12,13]. In China, several *Elsholtzia* species are commonly used as remedies for cough and cold [14]. Modern pharmacological studies revealed that plants of the *Elsholtzia* genus have many biological activities, including anti-inflammatory, anti-viral, and anti-bacterial activities [12,15,16]. *E. ciliata* is mainly distributed in East Asia, and *E. ciliata* is also used as a traditional remedy for common cold, fever, headache and diarrhea. In addition, *E. ciliata* ethanol extract has been reported to inhibit the replication of avian infectious bronchitis virus, indicating its potential as an antiviral agent [11]. However, the anti-coronaviral activities of *E. ciliata* extract have not been fully described.

Because coronavirus infection is associated with many common cold cases and *E. ciliata* has been used to treat the common cold, we examined whether *E. ciliata* extract showed antiviral activities to coronavirus. Here, we showed that the ethanol extract of *E. ciliata* can reduce coronavirus replication and virus-induced plaque formation. Moreover, we isolated the main signals of this plant into single components and compared the HPLC retention time of the identified compounds **1**–**4** with them. Among them, Luteolin-7-*O*-glucoside (**1**) was revealed to have the potential antiviral activity. In a previous study, luteolin-7-*O*-glucoside, one of the main flavonoids in *E. ciliata*, was reported to inhibit hepatitis B virus replication in vitro [17]. These findings support its potential as a candidate for antiviral drug development.

## Materials and methods

### Plant material

Aerial parts of *E. ciliata* were purchased from the herbal company (Kwangmyungdang, Ulsan, Korea). The sample was authenticated by Dr. J.H. Kim at the Department of Herbal Crop Research, National Institute of Horticultural and Herbal Science (NIHHS), Republic Korea. The voucher specimens of *E. ciliata* (EC2303–2) was deposited at the Laboratory of Natural Products Chemistry, NIHHS, Republic of Korea.

### Extract of the aerial parts of E. ciliate

Dried aerial parts of *E. ciliata* (4.0 g) were extracted two times with ethanol (40 mL, 3 h) under sonication. The ethanol solvent was evaporated under reduced pressure to obtain the ethanol residue (149.4 mg).

### Cell culture and infection

RD cells were cultured in DMEM (Welgene, Seoul, Korea) containing 10% FBS (Thermo Fisher Scientific, Waltham, MA, USA) and 1% penicillin-streptomycin solution (Welgene) at 37°C in a CO₂ incubator with 5% CO₂. The cells were washed twice with phosphate-buffered saline (PBS) and subsequently infected with HCoV-OC43 at a multiplicity of infection (MOI) of 0.01. The virus was diluted in MEM containing 2% FBS and co-incubated with either DMSO or ECE prior to cell infection. The infected cells were maintained at 33°C for 72 hours. Cell viability was measured using the MTT assay as described previously [18]. HCoV-OC43 virus was purchased from ATCC (Rockville, MD, USA), and RD cells were obtained from Korean Cell Line Bank (KCLB, Seoul, Korea). MTT was purchased from USB Corporation (Cleveland, OH, USA).

### Plaque formation assay

A plaque formation assay was performed to determine the viral titer. The virus stock was serially diluted 10-fold in MEM and inoculated into a 12-well plate. The infected cells were incubated at 33°C with 5% CO₂ for 1 hour to allow viral adsorption. After incubation, 200 µL of the inoculum was overlaid with a 0.3% agarose-containing MEM medium. The cells were then incubated at 33°C for 96 hours to allow plaque formation. The infected cells were fixed using a 4% paraformaldehyde solution. Viral plaques were visualized by staining with a 0.2% crystal violet solution.

### Western blot and quantitative RT-PCR

For Western blot, cells, and conditioned media were collected and lysed in cell lysis buffer (150mM NaCl, 50mM HEPES (pH 7.5), and 1% NP40) containing a protease inhibitor cocktail (Roche, Basel, Switzerland). Cell lysates and conditioned media were resolved by SDS-PAGE and transferred to immune-blot PVDF membrane filters (Bio-Rad, Hercules, CA, USA). Coronavirus proteins were detected with a 1:10,000 dilution of primary HCoV-OC43 antibody using an ECL system (Dogen, Seoul, Korea). The images were acquired using the ChemiDoc Imaging System (Bio-Rad). The HCoV-OC43 antibody was purchased from Sigma-Aldrich (Saint-Louis, MO, USA). For quantitative RT-PCR, cells and conditioned media were harvested, and RNA was extracted using TRIzol (Thermo Fisher Scientific) by the manufacturer’s instructions and then analyzed using the StepOnePlus Real-Time PCR System (Thermo Fisher Scientific). The primers for HCoV-OC43 M, N, and RDRP genes were described previously [6]. The sequences of the primers were as follows: The forward primer for HCoV-OC43 N was 5′-AGG ATG CCA CCA AAC CTC AG-3′, and the reverse primer was 5′-TGG GGA ACT GTG GGT CAC TA-3′. For HCoV-OC43 M, the forward primer was 5′-ACG GTC ACA ATA ATA CGC GGT-3′ and the reverse primer was 5′-GGG TTG ATG GCA GTC GGT AA-3′. For HCoV-OC43 RNA-dependent RNA polymerase (RdRp), the forward primer was 5′-GAG TGT AGA TGC CCG TCT CG-3′ and the reverse primer was 5′-TGT GGC ACA CGA CTA CCT TC-3′.

### Immunofluorescence imaging and SEM analysis

For immunostaining, cells were infected with HCoV-OC43 and treated with ECE. Forty-eight hours after infection, cells were fixed with 4% paraformaldehyde for 10 minutes at room temperature to inactivate the virus. After fixation, cells were permeabilized with 0.2% Triton X-100 in PBS for 20 minutes and subsequently blocked with 3% bovine serum albumin in PBS. The cells were then incubated with a 1:1000 dilution of HCoV-OC43 antibody in PBS, followed by staining with a 1:1000 dilution of Alexa Fluor 488–conjugated secondary antibody (Thermo Fisher Scientific). Finally, the stained slides were washed 3 times with PBS, stained with DAPI, and mounted in a mounting medium (Vector Laboratories, Burlingame, CA, USA). Images were analyzed with a Carl Zeiss LSM710 confocal microscope (Carl Zeiss, Oberkochen, Germany). For SEM analysis, RD cells were infected with HCoV-OC43 and prepared for imaging as previously described [5]. Briefly, cells were fixed in 2.5% glutaraldehyde for 1 hour, dehydrated using a graded ethanol series, air-dried, and coated with platinum. The image was captured using a Carl Zeiss SEM SUPRA 40 microscope (Carl Zeiss).

### HPLC analysis of ECE

The qualitative analysis of isolated compounds **1**–**4** was performed on the Agilent 1260 infinity Ⅱ LC system (Santa Clara, CA, USA). The mixture of four (**1**–**4**) and ECE was analyzed on an EclipsePlus C18 column (5 µm, 250 × 4.6 mm) with a mobile phase consisting of distilled water (0.1% formic acid, A) and acetonitrile (0.1% formic acid, B). The elution conditions were 1–40% for 30 min, 40–100% for 30.1–35 min, 100% for 35.1–40 min, and 10–10% for 40.1–45 min in order. The flow rate injection volume was 1mL/min and 10 µL, respectively.

### Statistical analysis

Plaque formation, protein expression, quantitative RT-PCR, and MTT were evaluated by a 2-tailed Student’s t-test using Excel software (Microsoft, Redmond, WA, USA). A p-value of 0.05 was considered significant. IC_50_ values were calculated by non-linear regression using a four-parameter logistic model (variable slope) in GraphPad Prism (version 9.5, GraphPad Software, CA, USA).

## Results

### *Elsholtzia ciliata* extract (ECE) treatment decreases coronavirus-induced plaque formation

**Fig 1:**
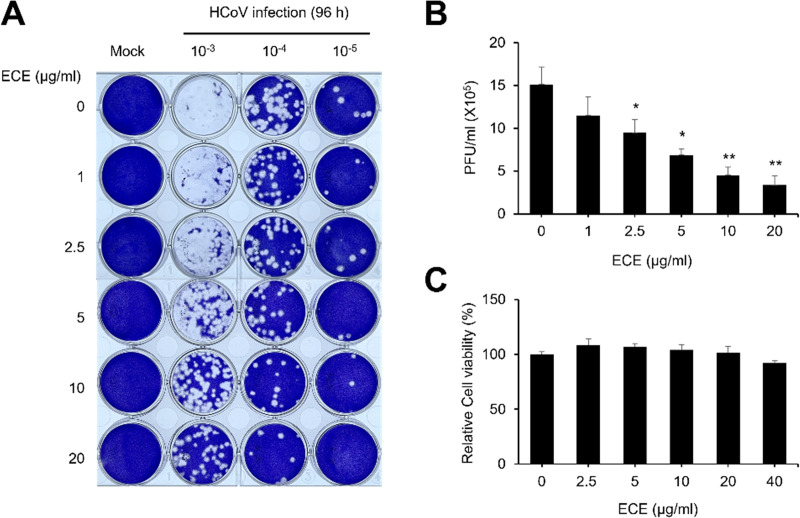
*Elsholtzia ciliata* extract (ECE) treatment decreases coronavirus-induced plaque formation. **(A)** Plaque formation assay with ECE. RD cells were treated with mock or ECE, and the infection media contained indicated dilutions of the HCoV-OC43 virus were replaced. Cells were overlayed 0.3% agarose, incubated for 4 days, and stained with crystal violets. **(B)** ECE treatment decreases plaque formation in a dose-dependent manner. The number of plaques was counted and shown in the graph. Mock vs ECE treatment (N = 4), *: *p* < 0.05, **: *p* < 0.01, ***: *p* < 0.005. **(C)** ECE did not have significant cytotoxicity up to 40 μg/ml. RD cells were incubated with either mock or the indicated concentration of ECE for 24 h, and the MTT assay was performed to evaluate the cytotoxicity.

We attempted to determine whether *E. ciliata* plant contains anti-viral compounds against coronavirus. First, we examined the cytotoxicity of ECE to determine the experimental concentrations. We found that ECE does not reduce the viability of RD cells up to 40 μg/mL, suggesting that ECE can be used at least up to 40 μg/ml (Fig 1C). Next, we used plaque formation assay with the Human Coronavirus OC43 strain (HCoV-OC43). RD cells were infected with HCoV-OC43 and checked whether the number of plaque formations was reduced by ECE treatment. ECE treatment significantly decreased the number of plaque formation (Figs 1A and 1B). These results indicate that ECE contains anti-viral compounds against coronavirus.

### ECE treatment decreases coronavirus protein expression

**Fig 2:**
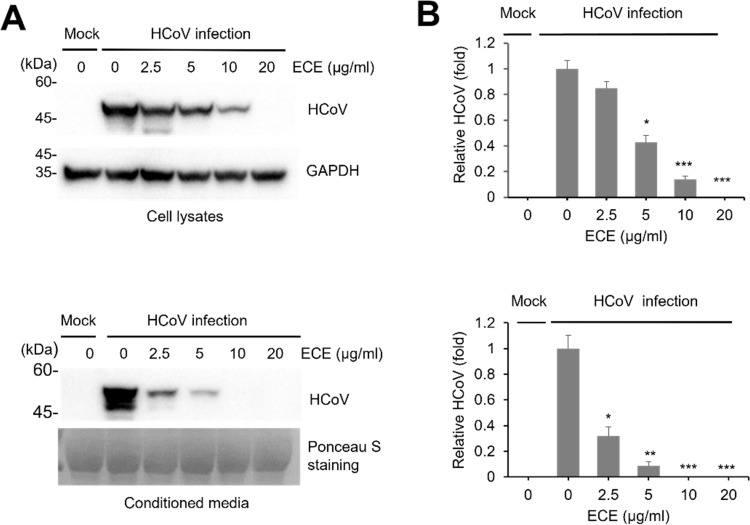
ECE treatment inhibits the expression of coronavirus proteins in a dose-dependent manner. **(A)** RD cells were infected with HCoV-OC43(10^−3^ dilution of conditioned media) and treated with ECE in 3 days. Cell lysates and the conditioned media were analyzed by Western blot with HCoV-OC43 antibody. GAPDH and ponceau S staining were used to control the loading control of cell lysates and conditioned media. **(B)** Coronavirus protein expressions were quantified using Western blot, and the graph shows the relative level. Control vs ECE treatment (N = 4), *: p < 0.05, **: p < 0.01, ***: p < 0.005.

Because ECE treatment decreased plaque formation, we examined whether ECE treatment can decrease viral protein expression. RD cells were infected with coronavirus, and cell lysates and conditioned media were collected and subjected to Western blot with anti-HCoV-OC43 antibody. ECE treatment significantly decreased coronavirus protein expression in cell lysates and conditioned media (Figs 2A and 2B). In addition, ECE treatment significantly decreased viral protein expression in the conditioned media at lower concentrations (2.5 μg/ml) (Fig 2B). These results indicate that ECE treatment decreased the protein expression of coronavirus.

### ECE treatment decreases coronavirus RNA copy number

**Fig 3:**
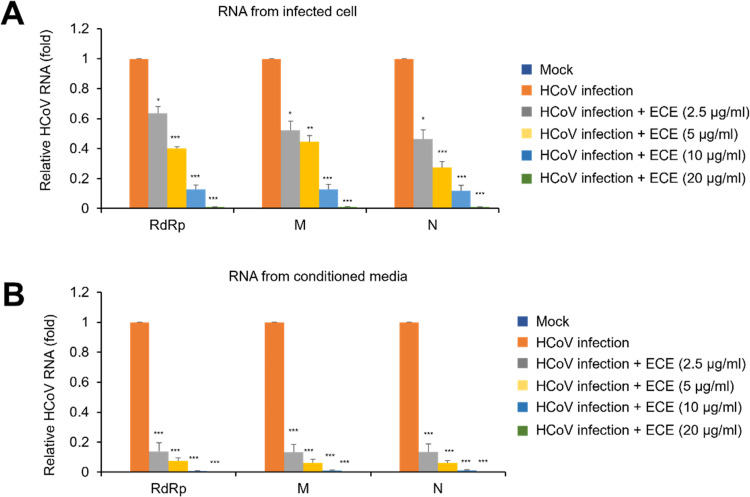
ECE decreases coronavirus RNA in a dose dependent manner. RD cells were infected with HCoV-OC43 and treated with the indicated concentration of ECE, and RNA was extracted from the cell (A) and the conditioned media **(B)**. Relative RNA expression levels of the RdRp gene, M gene, and N gene were evaluated by quantitative RT-PCR. Control vs ECE treatment (N = 3), *: *p* < 0.05, **: *p* < 0.01, ***: *p* < 0.005.

We examined whether ECE treatment can decrease viral RNA expression. Because coronavirus is a positive-strand RNA virus, the RNA levels in infected cells reflect both the viral genomic RNA and the subgenomic RNAs produced during infection. Total RNA was purified and quantified by quantitative RT-PCR. ECE treatment significantly decreased coronavirus RNA copy number in cell lysates and conditioned media (Figs 3A and 3B). Like protein expression, ECE treatment significantly reduced viral RNA copy number in the conditioned media at lower concentrations (2.5 μg/ml) (Fig 3B). These results indicate that ECE treatment decreased the RNA copy number of coronavirus. The IC_50_ values of ECE for inhibition of viral M, N, and RdRp gene expression were 3.12, 2.33, and 3.64 µg/mL, respectively. The average IC_50_ was calculated to be 3.03 ± 0.66 µg/mL (mean ± SD), based on non-linear regression using a four-parameter logistic model.

### ECE treatment decreases the number of infected cells

**Fig 4:**
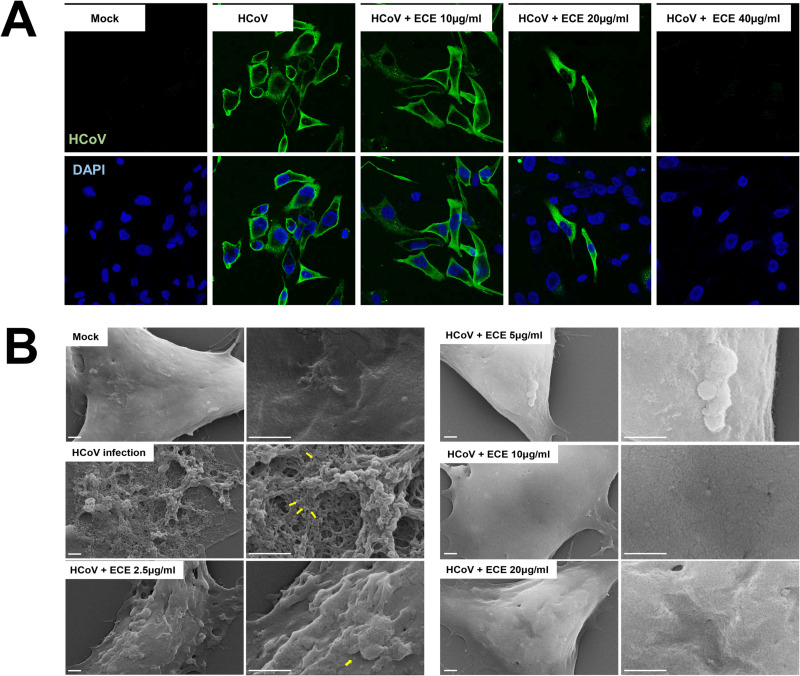
ECE treatment decreases coronavirus production in the cells. **(A)** ECE treatment decreased the coronavirus protein in the infected cells. RD cells were infected with HCoV-OC43 and treated with the indicated concentration of ECE. Cells were immune-stained with anti-HCoV-OC43 antibody. **(B)** ECE treatment decreased the coronavirus production. Scanning electron microscopy (SEM) images of RD cells infected with HCoV-OC43 and treated with ECE in 3 days. Scale bars, 1 μm.

Because ECE treatment decreased the level of viral protein and RNA, we next examined whether ECE treatment affects number of infected cells. RD cells were infected, stained with anti-HCoV-OC43 antibody, and observed by confocal microscopy. The number of infected cells was decreased by ECE treatment in a dose-dependent manner (Fig 4A). At 40 μg/ml of ECE treatment, we rarely detected the infected cells, and these results indicate that ECE treatment reduced the number of coronavirus-infected cells.

We attempted to detect the coronavirus production on the cell surface using SEM analysis. RD cells were infected and treated with ECE. Seventy-two hours after treatment, cells were fixed and examined with the scanning electron microscope (SEM). We observed the cytopathic effects and coronavirus production in the infected cells. However, ECE treatment decreased the coronavirus production and the cytopathic effect in a dose-dependent manner (Fig 4B). The cell surface of the infected cells with 10 µg/ml of ECE treatment showed a clear surface like a mock treatment (Fig 4B). These results indicate that ECE effectively reduces coronavirus production and cytopathic effects.

### HPLC analysis of ECE

**Fig 5:**
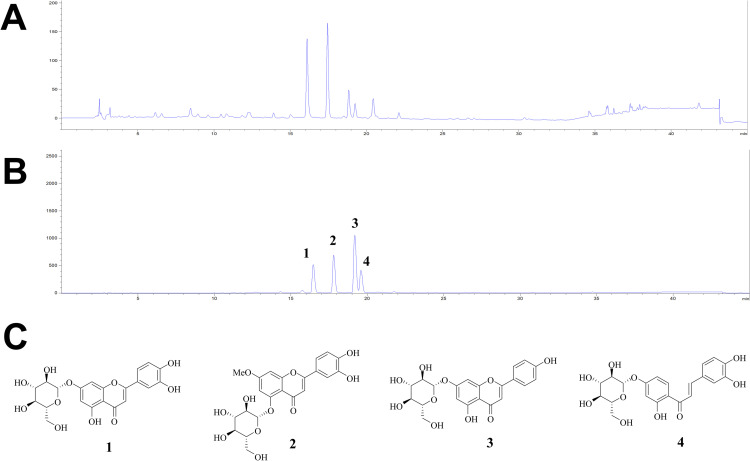
Analysis of ECE. ECE (A) and mixed compounds **1**–**4** (B) were analyzed by HPLC. The structure of isolated compounds **1**–**4 (C)** (**1**: luteolin-7-*O*-glucoside, **2**: Yuanhuanin, **3**: apigenin-7-*O*-glucoside, and **4**: butein-4′-*O*-glucoside).

The qualitative analysis of four chemical constitutions (**1**–**4**) in ECE was performed using HPLC with the gradient solvent system of distilled water (0.1% formic acid) and acetonitrile (0.1% formic acid). A mixture of the isolated compounds and ECE was tested with a C-18 column at 254 nm UV wavelength for 45 min. As shown in Figs. 5A and 5B, the primary signals of ECE were identified by comparing the retention times of the mixture (**1**: 15.095 min, **2**: 6.301 min, **3**: 17.735 min, and **4**: 18.117 min). Therefore, it was unveiled that luteolin-7-*O*-glucoside (**1**), Yuanhuanin (**2**), apigenin-7-*O*-glucoside (**3**), and butein-4′-*O*-glucoside (**4**) are main components in ECE (Fig 5C).

### Luteolin-7-*O*-glucoside is effective in inhibiting coronavirus replication

**Fig 6:**
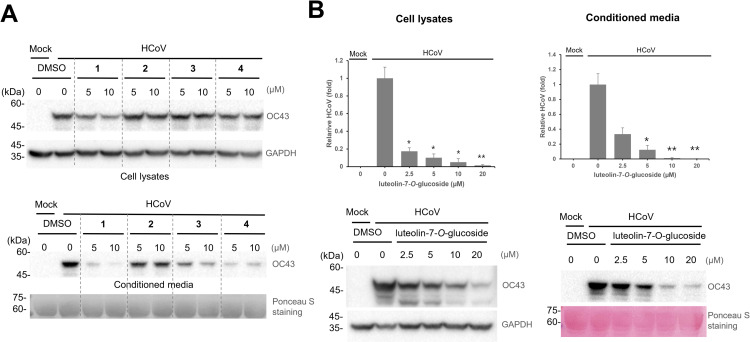
Luteolin-7-*O*-glucoside (1) is effective in inhibiting coronavirus replication. **(A)** The anti-viral effect of an isolated single compound was examined, and luteolin-7-*O*-glucoside (**1**) is most effective in reducing the expression of coronavirus protein. **(B)** Luteolin-7-*O*-glucoside (**1**) inhibits the coronavirus protein expression in the cell lysates and conditioned media. Control vs Luteolin-7-*O*-glucoside treatment (N = 4), *: *p* < 0.05, **: *p* < 0.01, ***: *p* < 0.005.

Because we isolated four main components of ECE, we examined whether they have antiviral effects. RD cells were infected with the coronavirus and treated with each single compound. Western blot with anti-HCoV antibody was used to evaluate the amount of coronavirus. We found that luteolin-7-*O*-glucoside (**1**), apigenin-7-*O*-glucoside (**3**), and butein-4′-*O*-glucoside (**4**) have antiviral effects, and the antiviral effects were more prominent in the condition (Fig 6A). Because the antiviral effect of compound (**1**) was remarkable, we repeated the experiment to confirm the results. The potential inhibitor (**1**) treatment reduced the coronavirus protein expression in a dose-dependent manner in the cell lysates and conditioned media (Fig 6B). The half inhibitory concentration of luteolin-7-*O*-glucoside (**1**) was less than 2.5 µM. Based on the dose-response data, the IC_50_ of luteolin-7-*O*-glucoside was estimated to be approximately 2µM (equivalent to 1 µg/mL), while IC_50_ of ECE was around 5 µg/mL, which is about five times higher than that of luteolin-7-*O*-glucosie. These findings suggest that luteolin-7-*O*-glucoside exhibits a greater antiviral effect than the extract.

## Discussions

This report demonstrated that *E. ciliata* extract has anti-viral activity against coronavirus. We used ethanol extract of *E. ciliata* and HCoV-OC43 virus to examine anti-viral activity. Because HCoV-OC43 and SARS-CoV-2 belong the same beta coronavirus, HCoV-OC43 is used as a less severe model of SARS-CoV-2 [7]. ECE treatment reduces the coronavirus-induced plaque formation and also inhibits the expression of coronavirus RNA and protein. Finally, we confirmed the reduction of coronavirus infection and production by microscopy. These results collectively indicate that the ethanol extract of *E. ciliata* has anti-viral activity.

Plants of the *Elsholtzia* genus and *E. ciliata* have been used as herbal medicine to treat the common cold and fever [12,15]. The common cold is mainly caused by the infection of respiratory viruses such as rhinovirus, adenovirus, and coronavirus [19,20]. Coronavirus infection accounts for 10 ~ 30% of the patients of common cold cases [1,2]. Therefore, we hypothesized that *E. ciliata* extract affects the replication of coronavirus. We performed several experiments to prove that ECE effectively inhibits coronavirus replication. Plaque formation assay is a gold standard for determining the concentration of infectious virus particles [21]. ECE treatment reduced the amount of plaque formation, indicating that ECE can reduce the infectious coronavirus (Fig 1). These results support that ECE is potentially applicable in treating coronavirus-related diseases. Next, we performed additional experiments to prove that ECE can inhibit coronavirus replication. Because HCoV-OC43 is commonly used to study SARS-CoV-2 as a less severe model system, the antibodies against HCoV-OC43 are available. The antibody against HCoV-OC43 can recognize the multiple proteins of coronavirus in Western blot, and we showed that ECE treatment decreased the number of coronavirus proteins in the infected cells (Fig 2). Interestingly, coronavirus proteins in the conditioned media are more dramatically reduced than cell lysates. Coronavirus proteins in the conditioned media are mainly derived from the coronavirus particles, and these results indicate that ECE treatment inhibited the release of coronavirus particles from the infected cells. Similar results were obtained from the RNA analysis of coronavirus (Fig 3). Quantitative RT-PCR showed that ECE treatment reduced coronavirus RNA both in cell lysates and conditioned media. Furthermore, the reduction in the conditioned media is more dramatic compared with cell lysates (Fig 3). These results collectively support that ECE treatment inhibits the coronavirus release from the infected cells.

We also examined the virus infection using microscopic analysis. Fluorescent microscopy data showed that the number of infected cells was reduced by ECE treatment in a dose-dependent manner (Fig 4A). A proportion of cells were infected with coronavirus first, and the rest of the cells were infected by the virus, which was amplified in the initially infected cells. Therefore, these data suggest that ECE treatment inhibits the formation of infectious coronavirus particles. Finally, SEM analysis also showed that ECE treatment interferes with the production of coronavirus (Fig 4B). We observed the apparent cytopathic effects and membrane protrusion in the coronavirus-infected cells. However, ECE treatment decreased the cytopathic effects dose-dependent (Fig 4B). Because the membrane deformation is related to coronavirus release [22], ECE treatment appears to interfere with the production and release of coronavirus. These results are consistent with the prominent reduction of coronavirus in the conditioned media (Fig 2B).

We analyzed the components of ECE and found that the main components of ECE are the glycoside derivatives of flavones (**1**–**3**) and chalcone (**4**) (Fig 5). Previous studies demonstrated that luteolin can potentially inhibit coronavirus replication and infection [23–25]. Therefore, we speculate that the luteolin-7-*O*-glucoside (**1**) in ECE inhibits coronavirus replication and infection. We also confirmed the inhibitory effects of these compounds by examining the coronavirus protein expression and found that luteolin-7-*O*-glucosdie is most effective in reducing the coronavirus proteins (Fig 6). Because ECE has luteolin derivatives, and these derivatives have antiviral effects, ECE is potentially useful for treating coronavirus-related infected diseases. Based on the dose-response data, the estimated IC50 values suggest that luteolin-7-*O*-glucoside (~2 µM; 1 µg/mL) exhibits greater antiviral activity than ECE (~5 µg/mL). Luteolin-7-*O*-glucoside appears to be one of key contributors to the antiviral activity of ECE and has potential as a lead compound for antiviral therapy. Further studies are needed to confirm its potential to treat coronavirus *in vivo*.

## Supporting information

S1 Fig Uncropped WB Image for Figure 2A. (PDF)

S2 Fig Uncropped WB Image for Figure 6B. (PDF)
