# Supplementary figures and images for: Luteolin-7-O-glucoside from Elsholtzia ciliata extract inhibits the replication of coronavirus
Source: PLoS One. 2025 Jun 3;20(6):e0325371. doi: 10.1371/journal.pone.0325371 (PMC12133163; doi:10.1371/journal.pone.0325371)

Figure 2A

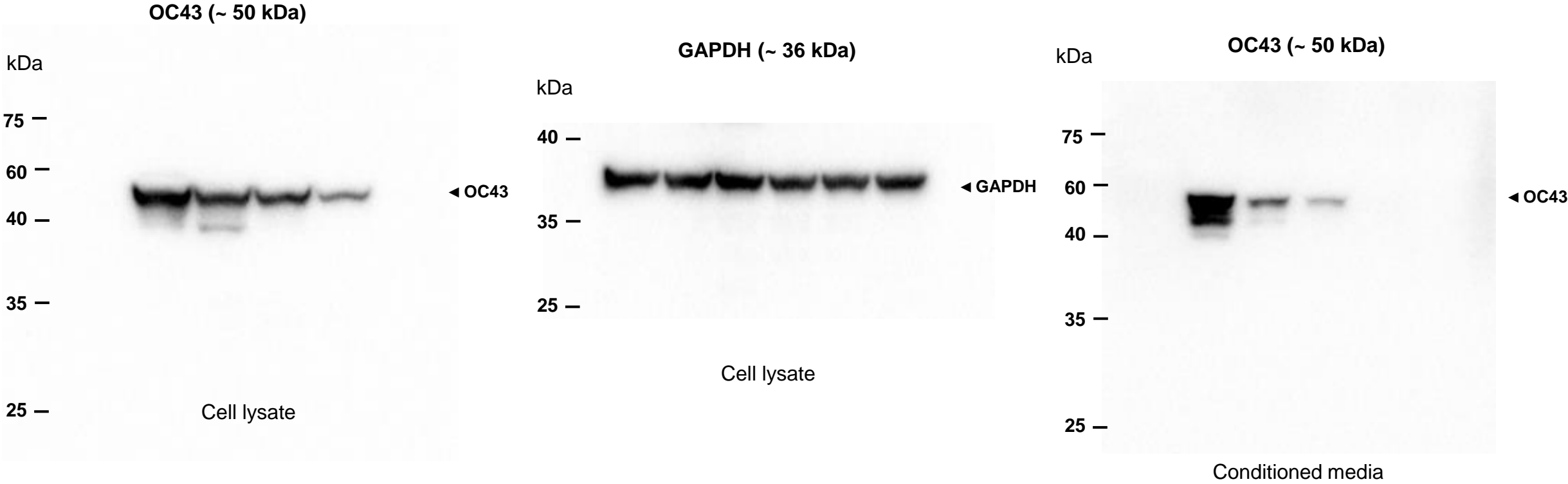

Supplement: S1 Fig — (PDF) [file pone.0325371.s001.pdf]

**Figure 6B**

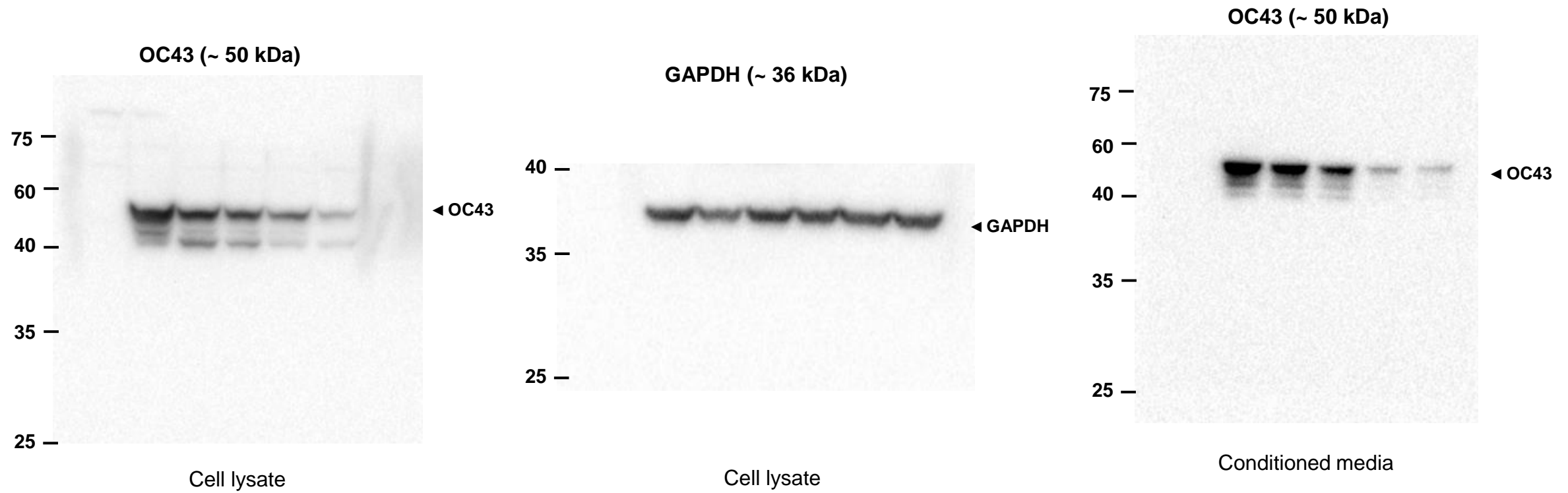

Supplement: S2 Fig — (PDF) [file pone.0325371.s002.pdf]
